# Kinematic Analysis of Exoskeleton-Assisted Community Ambulation: An Observational Study in Outdoor Real-Life Scenarios

**DOI:** 10.3390/s22124533

**Published:** 2022-06-16

**Authors:** Michela Goffredo, Paola Romano, Francesco Infarinato, Matteo Cioeta, Marco Franceschini, Daniele Galafate, Rebecca Iacopini, Sanaz Pournajaf, Marco Ottaviani

**Affiliations:** 1 IRCCS San Raffaele Roma, 00163 Rome, Italy; michela.goffredo@sanraffaele.it (M.G.); paola.romano@sanraffaele.it (P.R.); matteo.cioeta@sanraffaele.it (M.C.); marco.franceschini@sanraffaele.it (M.F.); daniele.galafate@sanraffaele.it (D.G.); rebecca.iacopini@gmail.com (R.I.); sanaz.pournajaf@sanraffaele.it (S.P.); marco.ottaviani@sanraffaele.it (M.O.); 2 Department of Human Sciences and Promotion of the Quality of Life, San Raffaele University, 00166 Rome, Italy

**Keywords:** benchmarking, gait, robotic exoskeleton, kinematics, lower extremity, community ambulation

## Abstract

(1) Background: In neurorehabilitation, Wearable Powered Exoskeletons (WPEs) enable intensive gait training even in individuals who are unable to maintain an upright position. The importance of WPEs is not only related to their impact on walking recovery, but also to the possibility of using them as assistive technology; however, WPE-assisted community ambulation has rarely been studied in terms of walking performance in real-life scenarios. (2) Methods: This study proposes the integration of an Inertial Measurement Unit (IMU) system to analyze gait kinematics during real-life outdoor scenarios (regular, irregular terrains, and slopes) by comparing the ecological gait (no-WPE condition) and WPE-assisted gait in five able-bodied volunteers. The temporal parameters of gait and joint angles were calculated from data collected by a network of seven IMUs. (3) Results: The results showed that the WPE-assisted gait had less knee flexion in the stance phase and greater hip flexion in the swing phase. The different scenarios did not change the human–exoskeleton interaction: only the low-speed WPE-assisted gait was characterized by a longer double support phase. (4) Conclusions: The proposed IMU-based gait assessment protocol enabled quantification of the human–exoskeleton interaction in terms of gait kinematics and paved the way for the study of WPE-assisted community ambulation in stroke patients.

## 1. Introduction

Walking independently in daily life is one of the most important functional tasks to regain after a stroke; however, up to 74% of stroke survivors suffer a disability in the Activities of Daily Living (ADLs) [1]: particularly upon discharge from an inpatient neurorehabilitation center, their independence in moving around at home or in the community is often significantly impaired [2]. Such poor autonomy in ADLs may result in depression and low Quality of Life (QoL), which in turn places a great economic burden on welfare and society [3]. Community ambulation is a complex activity that can be defined as “*independent mobility outside the home, which includes the ability to confidently deal with uneven terrain, private streets, rural roads, shopping centers, and public transportation*” [4]. Walking outdoors requires not only good motor control but also the cognitive ability to adapt motor control to various proprioceptive, auditory, and visual environmental stimuli (e.g., the type of terrain, the presence of sudden obstacles, and the ability to perform multiple attentional tasks) [3]. To achieve a good level of QoL at discharge, clinicians need to plan an appropriate and individualized rehabilitation program that should include intensive physical, occupational, and speech therapies [5,6]. Although epidemiological studies have shown that sociodemographic and clinical characteristics of subacute stroke survivors can predict good participation in walking activities in the community 6 months after stroke [7,8], walking recovery must be promoted by targeted intensive rehabilitation programs. In this context, Wearable Powered Exoskeletons (WPEs) could be valuable complementary rehabilitation devices, as they emulate overground human neuromotor control of locomotion, allowing early, intensive, and specific gait training even in individuals unable to maintain the upright position [9]. The efficacy of WPE-assisted gait training to improve motor and functional outcomes in stroke patients is well established in the literature [10,11,12,13,14,15,16,17,18], although the neurophysiological effects have not yet been adequately studied [19,20,21,22]. The relevance of WPEs for stroke patients is not only related to their effect on gait rehabilitation, but also to the possibility of using them as an assistive technology for walking in the community or at home settings [23]. Indeed, some WPEs are increasingly used outside hospitals to support walking in real-life [24,25]. Although the domestic use of WPE could have a strong impact on patients’ participation and QoL, it has rarely been evaluated in terms of walking performance in real-life scenarios [12,26]. In this context, the effect of WPE on locomotor patterns has yet to be characterized through experimental testing in real-life conditions with a standardized benchmarking framework [27,28].In this sense, the European EUROBENCH FSTP-2 subproject ESCALATE (ExoSkeleton assisted Community AmbuLAtion of healthy and sTrokE subjects) is investigating the use of a WPE in community ambulation of both healthy and stroke subjects to assess how the robotic solution meets the users’ needs, in terms of given assistance, performance indicators, safety, and user acceptance. 

This article aims to characterize the use of WPE outdoors in terms of walking performance. Gait kinematics in real-life outdoor scenarios (regular terrain, irregular terrain, and slopes) are analyzed by comparing ecological gait (no-WPE condition) and WPE-assisted gait in able-bodied volunteers. 

## 2. Materials and Methods

### 2.1. Study Design

This was an observational single-session-assessment pilot study to evaluate lower limb kinematics during the performance of gait tasks in various real-life outdoor scenarios. The study was conducted at the Neurorehabilitation Research Laboratory and Rehabilitation Bioengineering Laboratory of IRCCS San Raffaele Roma (Rome, Italy).

### 2.2. Participants

Adult able-bodied subjects who met the following criteria were recruited:

- Inclusion criteria:
  ○ age between 18–80 years;
  ○ ability to fit into the WPE (height between 155–195 cm; weight ≤ 113 kg; hip width ≤ 46 cm).
- Exclusion criteria:
  ○ persistent contractures of the joints;
  ○ orthopedic injuries;
  ○ pain;
  ○ severe osteoporosis;
  ○ skin breakdown;
  ○ pregnancy.

### 2.3. Experimental Setup

The WPE tested in the study was the Indego Therapy^®^ (Parker Hannifin Corp., Cleveland, OH, USA), an FDA-approved lower-limb powered exoskeleton marketed as a gait trainer and assistive device for use in clinical practice [29]. The Indego Therapy^®^ hardware consists of four motors for powered movement of bilateral hip and knee joints in the sagittal plane and built-in ankle-foot-orthoses at both ankle joints to provide stability and transfer the weight of the exoskeleton to the ground. Built-in electronic sensors include encoders at each joint that provide the respective joint angles and angular velocities and a six-axis inertial measurement unit in each thigh limb that provides the left and right thigh angles with respect to the vertical. The total mass of the exoskeleton, including the battery, is 17.7 kg. Indego Therapy^®^ can be disassembled and transported in a suitable external case: Each component is adjustable in length according to the anthropometric measurements of the user. The Motion+ software modality (100% assistance) was applied in the experimental protocol: The subject leans forward to initiate motion and Indego Therapy^®^ responds accordingly. The technology readiness level of Indego Therapy^®^ is 9 according to the European Union regulations. 

**Figure 1:**
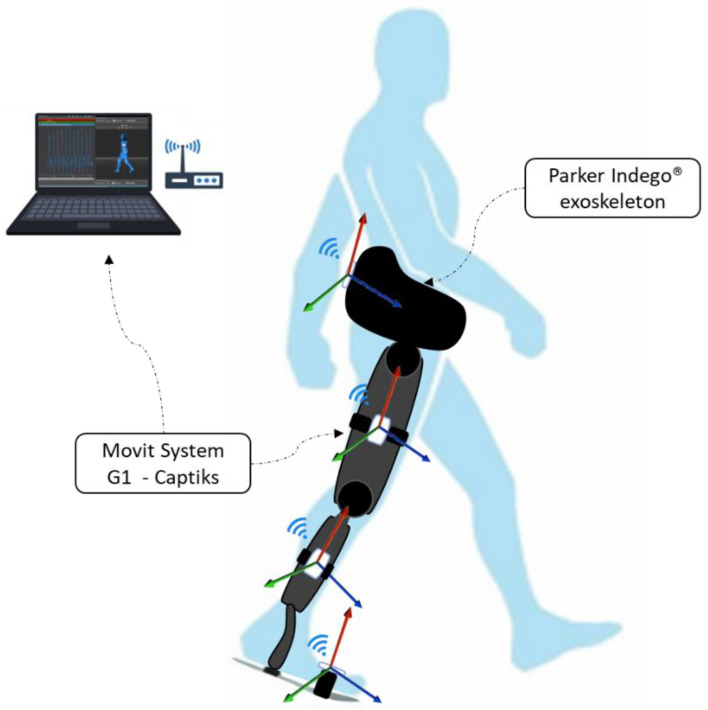
Experimental setup during WPE-assisted gait.

The MOVIT G1 Inertial Measurement Units (IMUs) system (Captiks srl, Rome, Italy) was used for motion capture [30]. Data were acquired at a frequency of 60 Hz. After the calibration phase of the IMU sensors network, seven IMUs were placed at the following points using elastic bands attached with Velcro strip on the following points: (1) at the level of the second sacral vertebra; (2) right thigh: in the middle of the segment connecting the right great trochanter and the right femoral condyles; (3) left thigh: at the midpoint of the segment connecting the right great trochanter and the right femoral condyle; (4) right lower leg: at the midpoint of the segment connecting the right femoral condyle and the right malleolus; (5) left lower leg: at the midpoint of the segment connecting the left femoral condyle and the left malleolus; (6) right foot: at the right metatarsal bones; (7) left foot: at the left metatarsal bones. In the ecological gait (no-WPE), the IMU’s elastic bands were applied to the subject’s skin, while in the WPE-assisted gait, they were applied to the WPE (see Figure 1).

### 2.4. Scenarios

Experiments were conducted in the following benchmarking scenarios defined by the European EUROBENCH FSTP-1 subprojects:

- Walking over regular terrain: walking through an indoor regular 10 m terrain (marble) at a self-selected normal pace.
- Walking over irregular terrain: walking through an irregular 10 m outdoor terrain (cobblestones) at self-selected normal speed, self-selected high speed, and self-selected low speed.
- Ascending slopes: walking on an 8 m outdoor ascending ramp (standard inclined wheelchair ramp; slope 8%) at self-selected normal speed.
- Descending slopes: walking on an 8 m outdoor descending ramp (standard inclined wheelchair ramp; slope 8%) at a self-selected normal speed.

**Figure 2:**
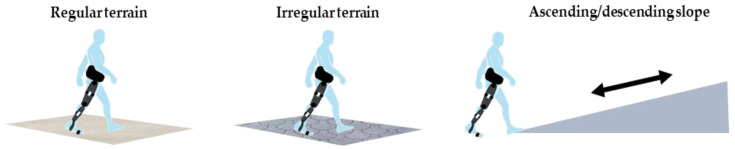
Schematic of scenarios: regular indoor terrain, irregular outdoor terrain, and ascending/descending slope.

For each scenario, two walking conditions are considered: (1) ecological gait (no-WPE); (2) WPE-assisted gait. Four walking trials were performed under each condition to ensure the consistency of data. A 10-min rest was allowed between conditions to minimize fatigue. A schematic of the scenarios is depicted in Figure 2.

### 2.5. Data Processing

The Captiks Motion Analyzer software provided the joint angles in the sagittal, frontal, and transverse planes and the calibrated quaternions. The angles were analyzed using in-house software developed with MATLAB R2020a (The MathWorks, Natick MA, USA), the heel strike and toe-off were extracted, and the following temporal gait parameters were calculated:

- Cadence (step/min)—number of steps taken in one-minute time;
- Gait cycle (s)—the mean duration of the gait cycle starting with the first heel contact and ending with the next heel contact of the same limb;
- Double support (s)—time during which the feet are on the ground;
- Stance (s)—time starting with the first contact and ending at the toe-off of the same limb;
- Swing (s)—time beginning with toe-off and ending with the first contact of the same limb.

Joint kinematics were normalized as a percentage of the gait cycle, producing kinematic plots of the following parameters: hip flexion/extension; hip abduction/adduction; knee flexion/extension; knee rotation; ankle dorsiflexion/plantar flexion; foot progression.

All calculated parameters were averaged within subjects for each scenario. Considering the observational pilot nature of the study, descriptive statistics were performed on gait parameters and joint angles.

### 2.6. Ethical Aspects

This study was conducted in accordance with the Declaration of Helsinki and was approved by the local ethics committee (No. PR 21/05, dated 28 April 2021). Participants were enrolled in the study after signing an informed consent form.

## 3. Results

Five able-bodied subjects were enrolled and participated in all experimental scenarios described in the previous paragraph. Table 1 depicts the demographical and anthropometrical data of the recruited subjects. The WPE was adjusted to the anthropometric features of each subject.

All participants performed the gait tasks without difficulty. The IMU sensors network recorded the movement during the gait with and without the WPE. The temporal parameters of gait and joint angles were calculated.

Table 2 shows the temporal gait parameters computed for each scenario and each walking condition. The data for the no-WPE condition are consistent with those of healthy subjects from the literature [4]. The calculated gait parameters are similar in all experimental scenarios when subjects performed the ecological gait. The WPE-assisted gait was characterized by a lower cadence (with a maximum value of 28.00 steps/min) than the self-selected gait without WPE, and the duration of the gait cycle is also higher in the WPE-assisted gait than in ecological gait. The duration of double support, stance, and swing was consistent with normative data. Differences between the gait conditions were registered in the irregular terrain in a low-speed scenario: the average time for the double support (as a percentage of the gait cycle) was 68.31% and 52.72% in the WPE condition and no-WPE condition, respectively. Thus, the WPE-assisted slow gait was characterized by a higher double support phase. No differences were observed in the other scenarios. The stance phase (as a percentage of the gait cycle) was always higher (about 60%) in the WPE condition compared to the ecological gait. 

Joint kinematics (normalized as a percentage of the gait cycle) were depicted during the ecological gait and the WPE-assisted gait in the regular indoor terrain, descending, and ascending slope scenarios in Figure 2. Although temporal gait parameters did not show great inter-scenarios differences, the joint angles revealed some variations, especially in the no-WPE conditions. In the ecological gait, the ascending slope scenario was characterized by higher hip flexion and smaller knee and ankle flexions compared to the regular indoor terrain and descending slope scenarios. On the other hand, joint kinematics during the WPE-assisted gait tasks showed a strong similarity. 

When comparing the gait states, it is noticeable that the WPE performed a significant hip flexion during the swing phase. In addition, the knee flexion and extension angle showed an atypical trend when the subjects wore the WPE. Specifically, a large difference in the amount of flexion during the loading response phase occurred. Normally, the knee should flex rapidly during the loading phase after the onset of the stance phase: flexion during phase flexion should be around 15°/18° and corresponds to the phase in which the knee is loaded maximally. In the WPE condition, on the other hand, the knee angle in the loading phase is approximately zero. It is important to note that such an atypical trend is highly visible in all scenarios. The ankle joint was limited in its flexion and extension movements during the WPE-assisted gait compared to the ecological gait. A similar restriction was registered in the abduction and adduction movements of the hip under the same gait conditions.

**Figure 3:**
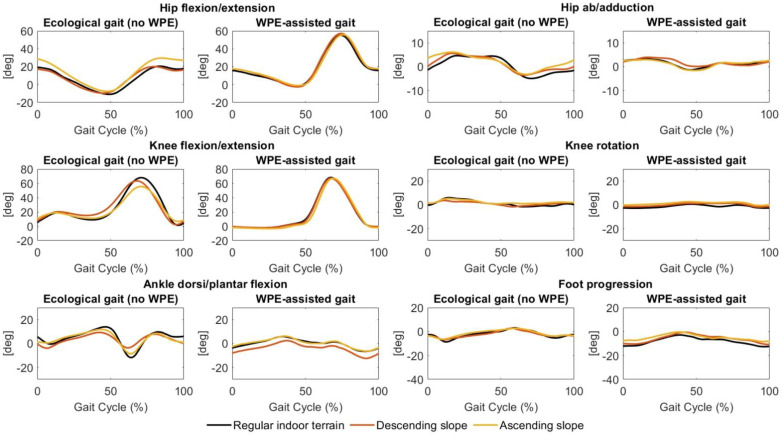
Joint kinematics (normalized as a percentage of the gait cycle) during ecological gait (no-WPE) and WPE-assisted gait in the regular indoor terrain, descending slope, and ascending slope scenarios.

**Figure 4:**
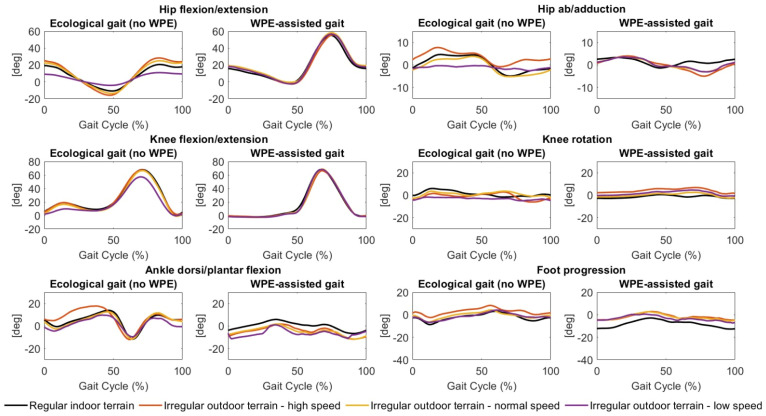
Joint kinematics (normalized as a percentage of the gait cycle) during ecological gait (no-WPE) and WPE-assisted gait in the regular indoor terrain and irregular outdoor terrain (high speed; normal speed; and low speed) scenarios.

Figure 3 and Figure 4, and Table 3 show joint kinematics and range of execution, respectively, during ecological gait and WPE-assisted gait under regular indoor terrain, descending slope, ascending slope scenarios, and irregular outdoor terrain (high speed, normal speed, and low speed) scenarios. In ecological gait, the low-speed irregular scenario was characterized by a limited range of execution in hip flexion/extension and abduction/adduction movements. In the irregular terrain scenarios, joint kinematics during WPE-assisted gait tasks showed a strong similarity. Similarly, to the previously described scenarios, the WPE-assisted gait is characterized by greater hip flexion during the swing phase and no-knee flexion during the loading response phase.

## 4. Discussion

This study was conducted as part of the European EUROBENCH FSTP-2 subproject ESCALATE (ExoSkeleton assisted Community AmbuLAtion of healthy and sTrokE subjects) as the WPE-assisted walking performance has rarely been evaluated in real-life scenarios with a standardized benchmarking framework [27]. Other studies focused on the kinematics of able-bodied subjects and neurological patients during WPE-assisted walking in a controlled environment (10- or 12-m walkway, treadmill, with or without weight suspension) [17,31,32,33]. In our study, gait kinematics in real-life outdoor scenarios (regular terrain, irregular terrains, and slopes) were analyzed by comparing ecological gait and WPE-assisted gait in five healthy volunteers. Participants were uninjured and showed gait data consistent with typical gait without WPE. 

Similar to Swank et al. [26], the WPE-assisted community ambulation showed differences from typical walking in terms of temporal gait parameters and joint movements. Participants in this study walked with the WPE at approximately half their average cadence in all scenarios considered. Nevertheless, the double-limb support time was similar in all scenarios (approximately 60% of the gait cycle) except for the irregular outdoor terrain at low speed. Specifically, in the latter scenario, an increase in double-limb support time was found when the participants wore the WPE. We observed several changes in joint kinematics when walking with WPE as compared to ecological gait. The outcomes are consistent with the study by Swank et al. [26], who analyzed walking with a robotic exoskeleton in healthy subjects and found that the restricted ankle motion in the WPE-supported situation could be overcome by greater hip flexion during the swing phase.

The inter-scenario analysis did not reveal any relevant differences in joint kinematics during WPE-assisted gait: the able-bodied subjects wearing the WPE walked with a similar gait pattern. This outcome is to be expected since the Motion+ software modality (100% support) was applied in the experimental protocol with the Indego Therapy^®^ WPE.

Some aspects of the study limited the generalizability of our conclusions. First, the sample size was small and consisted of young, healthy, and active individuals. Future studies with elderly and/or stroke patients may help to better understand the performance of WPE-induced gait. Second, the study did not estimate the 3D trajectories of the lower limbs. Analyzing the movement of each body segment during walking in each scenario could help characterize locomotion, especially in complex terrain or perturbations. Third, Indego therapy^®^ settings were not modified in the study (only gait speed was changed in accordance with the scenario’s description). A gait kinematics-based fine-tuning procedure for the automatic setting of WPE parameters could be on the future research agenda to tailor the use of the WPE in community ambulation. 

## 5. Conclusions

The use of WPE for gait in real-life outdoor scenarios is feasible, safe, and allows the user to walk overground with a typical smooth gait pattern. Compared to the ecological walking, the WPE-assisted one showed less knee flexion and greater hip flexion in the stance and swing phases, respectively. The different scenarios did not change the human–exoskeleton interaction. The WPE increased the double support phase during the low-speed gait tasks. Future studies of the use of a WPE in community ambulation of both healthy and stroke subjects are needed to quantify the human–exoskeleton interaction from the robotic, biomechanical, and physiological perspectives and to assess gait kinematics with and without WPE in stroke patients. Furthermore, it would be of strong interest the analysis of kinematics during dual-task walking with and without using a WPE.

Regarding the scenarios, further studies are also needed to explore different real-life scenarios, such as outdoor streets, sidewalks, and different irregular terrains.

## Figures and Tables

**Table 1:** Characteristics of the enrolled subjects.

	Gender	Age	Height (cm)	Weight (kg)
Subj A	M	26	187	74
Subj B	F	33	160	58
Subj C	F	24	172	60
Subj D	F	43	163	56
Subj E	M	27	172	72

**Table 2:** Gait temporal parameters calculated for each gait condition (WPE: WPE-assisted gait; no-WPE: ecological gait without WPE) in each scenario.

		Regular Terrain			Irregular Terrain		
		Indoor	Descending Slope	Ascending Slope	High Speed	Normal Speed	Low Speed
**cadence (step/min)**	WPE	22.02 ± 0.55	23.30 ± 0.25	22.38 ± 0.41	26.31 ± 0.7	22.65 ± 0.33	19.90 ± 0.32
	no-WPE	52.66 ± 1.69	50.74 ± 2.4	48.84 ± 1.98	59.76 ± 4.13	49.72 ± 2.20	41.85 ± 4.95
**gait cycle (s)**	WPE	2.75 ± 0.07	2.59 ± 0.02	2.70 ± 0.04	2.30 ± 0.06	2.66 ± 0.03	3.03 ± 0.04
	no-WPE	1.16 ± 0.04	1.21 ± 0.06	1.25 ± 0.04	1.04 ± 0.08	1.23 ± 0.05	1.65 ± 0.38
**Double** **support (×100 s)**	WPE	1.89 ± 0.06	1.79 ± 0.04	1.77 ± 0.10	1.51 ± 0.04	1.78 ± 0.06	2.07 ± 0.03
	no-WPE	0.83 ± 0.02	0.84 ± 0.06	0.83 ± 0.08	0.70 ± 0.08	0.83 ± 0.03	0.87 ± 0.09
**stance (s)**	WPE	1.57 ± 0.06	1.45 ± 0.03	1.55 ± 0.08	1.34 ± 0.06	1.55 ± 0.05	1.73 ± 0.04
	no-WPE	0.59 ± 0.01	0.58 ± 0.03	0.64 ± 0.05	0.53 ± 0.05	0.62 ± 0.01	0.67 ± 0.10
**swing (s)**	WPE	1.17 ± 0.04	1.13 ± 0.03	1.14 ± 0.09	0.95 ± 0.02	1.1 ± 0.02	1.29 ± 0.03
	no-WPE	0.56 ± 0.04	0.63 ± 0.05	0.6 ± 0.04	0.51 ± 0.03	0.61 ± 0.05	0.98 ± 0.29

Abbreviations: WPE: WPE-assisted gait; no-WPE: ecological gait without WPE.

**Table 3:** Range of execution of joint kinematics calculated for each gait condition (WPE: WPE-assisted gait; no-WPE: ecological gait without WPE) in each scenario.

			Regular Terrain			Irregular Terrain		
			Indoor	Descending Slope	Ascending Slope	High Speed	Normal Speed	Low Speed
			Min, Max	Min, Max	Min, Max	Min, Max	Min, Max	Min, Max
**Hip**	**A-A**	WPE	−6.26, 6.49	−3.21, 5.28	−4.99, 5.86	−5.49, 3.95	−4.24, 4.00	−4.02, 4.59
		no-WPE	−10.52, 8.01	−5.10, 7.47	−5.05, 8.12	−4.46, 10.27	−9.99, 6.48	−8.5, 8.14
	**F-E**	WPE	−2.79, 57.11	−2.39, 58.42	−2.24, 58.57	−1.74, 60.16	−1.51, 59.56	−0.36, 59.20
		no-WPE	−10.54, 25.02	−12.22, 24.47	−11.4, 32.47	−13.02, 34.4	−12.83, 27.78	−11.5, 17.39
**Knee**	**F-E**	WPE	−2.43, 71.72	−1.14, 71.18	−3.6, 72.58	−3.60, 69.99	−3.23, 70.72	−3.33, 70.86
		no-WPE	−0.62, 69.63	−2.02, 71.52	−1.92, 60.6	−2.88, 63.37	−3.03, 67.7	−5.39, 60.63
	**R**	WPE	0.64, 7.54	−2.66, 5.91	−2.49, 5.92	−1.38, 8.79	−4.11, 4.81	−0.93, 7.53
		no-WPE	−8.58, 8.50	−8.68, 7.16	−6.76, 10.14	−25.55, −6.23	−8.00, 9.75	−4.96, 13.53
**Ankle**	**DPF**	WPE	−9.59, 7.60	−13.44, 4.29	−9.89, 10.70	−16.83, 6.36	−13.32, 5.15	−11.92, 2.63
		no-WPE	−16.27, 15.11	−11.5, 14.59	−13.69, 15.20	−14.65, 16.95	−13.44, 14.32	−17.36, 14.55
	**FP**	WPE	−11.84, 1.30	−9.24, 2.61	−14.92, 2.73	−9.78, 5.31	−8.85, 3.73	−8.52, 2.09
		no-WPE	−11.05, 5.75	−6.98, 4.86	−9.55, 4.14	6.73, 21.50	−8.66, 5.67	−5.58, 11.22

Abbreviations: A-A: Abduction-adduction; F-E: Flexion-extension; R: Rotation; DPF: Dorsi plantar flexion; FP: Foot progression; WPE: WPE-assisted gait; no-WPE: ecological gait without WPE.

## Data Availability

The data presented in this study are available on request from the corresponding author.

## References

[1] Miller E.L., Murray L., Richards L., Zorowitz R.D., Bakas T., Clark P., Billinger S.A., American Heart Association Council on Cardiovascular Nursing and the Stroke Council (2010). Comprehensive overview of nursing and interdisciplinary rehabilitation care of the stroke patient: A scientific statement from the American Heart Association. Stroke.

[2] Balasubramanian C.K., Clark D.J., Fox E.J. (2014). Walking adaptability after a stroke and its assessment in clinical settings. Stroke Res. Treat..

[3] Pellicciari L., Agosti M., Goffredo M., Pournajaf S., Le Pera D., De Pisi F., Franceschini M., Damiani C. (2021). Factors Influencing Functional Outcome at Discharge: A Retrospective Study on a Large Sample of Patients Admitted to an Intensive Rehabilitation Unit. Am. J. Phys. Med. Rehabil..

[4] Perry J., Garrett M., Gronley J.K., Mulroy S.J. (1995). Classification of walking handicap in the stroke population. Stroke.

[5] Barbeau H. (2003). Locomotor training in neurorehabilitation: Emerging rehabilitation concepts. Neurorehabilit. Neural Repair.

[6] Seccia R., Boresta M., Fusco F., Tronci E., Di Gemma E., Palagi L., Mangone M., Agostini F., Bernetti A., Santilli V. (2020). Data of patients undergoing rehabilitation programs. Data Brief.

[7] Pournajaf S., Goffredo M., Agosti M., Massucci M., Ferro S., Franceschini M., Italian Study Group on Implementation of Stroke Care (ISC Study) (2019). Community ambulation of stroke survivors at 6 months follow-up: An observational study on sociodemographic and sub-acute clinical indicators. Eur. J. Phys. Rehabil. Med..

[8] Selves C., Stoquart G., Lejeune T. (2020). Gait rehabilitation after stroke: Review of the evidence of predictors, clinical outcomes and timing for interventions. Acta Neurol. Belg..

[9] Vale N., Gandolfi M., Vignoli L., Botticelli A., Posteraro F., Morone G., Dell’Orco A., Dimitrova E., Gervasoni E., Goffredo M. (2021). Electromechanical and Robotic Devices for Gait and Balance Rehabilitation of Children with Neurological Disability: A Systematic Review. Appl. Sci..

[10] Gandolfi M., Vale N., Posteraro F., Morone G., Dell’orco A., Botticelli A., Dimitrova E., Gervasoni E., Goffredo M., Zenzeri J. (2021). State of the art and challenges for the classification of studies on electromechanical and robotic devices in neurorehabilitation: A scoping review. Eur. J. Phys. Rehabil. Med..

[11] Molteni F., Guanziroli E., Goffredo M., Calabro R.S., Pournajaf S., Gaffuri M., Gasperini G., Filoni S., Baratta S., Galafate D. (2021). Gait Recovery with an Overground Powered Exoskeleton: A Randomized Controlled Trial on Subacute Stroke Subjects. Brain Sci..

[12] Pinto-Fernandez D., Torricelli D., Sanchez-Villamanan M.D., Aller F., Mombaur K., Conti R., Vitiello N., Moreno J.C., Pons J.L. (2020). Performance Evaluation of Lower Limb Exoskeletons: A Systematic Review. IEEE Trans. Neural Syst. Rehabil. Eng..

[13] Goffredo M., Guanziroli E., Pournajaf S., Gaffuri M., Gasperini G., Filoni S., Baratta S., Damiani C., Franceschini M., Molteni F. (2019). Overground wearable powered exoskeleton for gait training in subacute stroke subjects: Clinical and gait assessments. Eur. J. Phys. Rehab. Med..

[14] Goffredo M., Iacovelli C., Russo E., Pournajaf S., Di Blasi C., Galafate D., Pellicciari L., Agosti M., Filoni S., Aprile I. (2019). Stroke Gait Rehabilitation: A Comparison of End-Effector, Overground Exoskeleton, and Conventional Gait Training. Appl. Sci..

[15] Morone G., Paolucci S., Cherubini A., De Angelis D., Venturiero V., Coiro P., Iosa M. (2017). Robot-assisted gait training for stroke patients: Current state of the art and perspectives of robotics. Neuropsychiatr. Dis. Treat..

[16] Hsu T.H., Tsai C.L., Chi J.Y., Hsu C.Y., Lin Y.N. (2022). Effect of wearable exoskeleton on post-stroke gait: A systematic review and meta-analysis. Ann. Phys. Rehabil. Med..

[17] Taki S., Iwamoto Y., Imura T., Mitsutake T., Tanaka R. (2022). Effects of gait training with the Hybrid Assistive Limb on gait ability in stroke patients: A systematic review of randomized controlled trials. J. Clin. Neurosci..

[18] Lorusso M., Tramontano M., Casciello M., Pece A., Smania N., Morone G., Tamburella F. (2022). Efficacy of Overground Robotic Gait Training on Balance in Stroke Survivors: A Systematic Review and Meta-Analysis. Brain Sci..

[19] Infarinato F., Romano P., Goffredo M., Ottaviani M., Galafate D., Gison A., Petruccelli S., Pournajaf S., Franceschini M. (2021). Functional Gait Recovery after a Combination of Conventional Therapy and Overground Robot-Assisted Gait Training Is Not Associated with Significant Changes in Muscle Activation Pattern: An EMG Preliminary Study on Subjects Subacute Post Stroke. Brain Sci..

[20] Goffredo M., Infarinato F., Pournajaf S., Romano P., Ottaviani M., Pellicciari L., Galafate D., Gabbani D., Gison A., Franceschini M. (2020). Barriers to sEMG Assessment During Overground Robot-Assisted Gait Training in Subacute Stroke Patients. Front. Neurol..

[21] Tamburella F., Tagliamonte N.L., Pisotta I., Masciullo M., Arquilla M., van Asseldonk E.H.F., van der Kooij H., Wu A.R., Dzeladini F., Ijspeert A.J. (2020). Neuromuscular Controller Embedded in a Powered Ankle Exoskeleton: Effects on Gait, Clinical Features and Subjective Perspective of Incomplete Spinal Cord Injured Subjects. IEEE Trans. Neural. Syst. Rehabil. Eng..

[22] Afzal T., Zhu F., Tseng S.C., Lincoln J., Francisco G., Su H., Chang S.H. (2022). Evaluation of Muscle Synergy during Exoskeleton-assisted Walking in Persons with Multiple Sclerosis. IEEE Trans. Biomed. Eng..

[23] Raab K., Krakow K., Tripp F., Jung M. (2016). Effects of training with the ReWalk exoskeleton on quality of life in incomplete spinal cord injury: A single case study. Spinal Cord. Ser. Cases.

[24] Bissolotti L., Nicoli F., Picozzi M. (2018). Domestic Use of the Exoskeleton for Gait Training in Patients with Spinal Cord Injuries: Ethical Dilemmas in Clinical Practice. Front. Neurosci..

[25] Bayon C., Delgado-Oleas G., Avellar L., Bentivoglio F., Di Tommaso F., Tagliamonte N.L., Rocon E., van Asseldonk E.H.F. (2021). Development and Evaluation of BenchBalance: A System for Benchmarking Balance Capabilities of Wearable Robots and Their Users. Sensors.

[26] Swank C., Wang-Price S., Gao F., Almutairi S. (2019). Walking With a Robotic Exoskeleton Does Not Mimic Natural Gait: A Within-Subjects Study. JMIR Rehabil. Assist. Technol..

[27] Torricelli D., Rodriguez-Guerrero C., Veneman J.F., Crea S., Briem K., Lenggenhager B., Beckerle P. (2020). Benchmarking Wearable Robots: Challenges and Recommendations From Functional, User Experience, and Methodological Perspectives. Front. Robot. AI.

[28] Torricelli D., Veneman J., Gonzalez-Vargas J., Mombaur K., Remy C.D. (2019). Editorial: Assessing Bipedal Locomotion: Towards Replicable Benchmarks for Robotic and Robot-Assisted Locomotion. Front. Neurorobot..

[29] Tefertiller C., Hays K., Jones J., Jayaraman A., Hartigan C., Bushnik T., Forrest G.F. (2018). Initial Outcomes from a Multicenter Study Utilizing the Indego Powered Exoskeleton in Spinal Cord Injury. Top Spinal Cord Inj. Rehabil..

[30] Saggio G., Tombolini F., Ruggiero A. (2021). Technology-Based Complex Motor Tasks Assessment: A 6-DOF Inertial-Based System Versus a Gold-Standard Optoelectronic-Based One. IEEE Sens. J..

[31] Hayes S.C., White M., Wilcox C.R.J., White H.S.F., Vanicek N. (2022). Biomechanical differences between able-bodied and spinal cord injured individuals walking in an overground robotic exoskeleton. PLoS ONE.

[32] Park J.H., Kim S., Nussbaum M.A., Srinivasan D. (2022). Effects of back-support exoskeleton use on gait performance and stability during level walking. Gait Posture.

[33] Laubscher C.A., Goo A., Farris R.J., Sawicki J.T. (2022). Hybrid Impedance-Sliding Mode Switching Control of the Indego Explorer Lower-Limb Exoskeleton in Able-Bodied Walking. J. Intell. Robot. Syst..

